# Access to palliative care in India: situational analysis and modeling of access from public healthcare centers

**DOI:** 10.3332/ecancer.2025.2038

**Published:** 2025-11-19

**Authors:** Parth Sharma, Harsh Thakkar, Aryan Patil, Preeti Chauhan, Priya Chembon, Shalini Arora Joseph, Smriti Rana, Raj Kalady, Vidhi Wadhwani, Gaurav Urs, Padmavathy Krishna, Rontu Sangma, Rajendra Dutt Bijalwan, Sunanda Samal, Lalit Selvaraju, Syed Mohammad Askari Naqvi, Jatin Bhukal, Johnsurya John, Muttacaud Ramakrishnan Rajagopal, Siddhesh Zadey

**Affiliations:** 1 Association for Socially Applicable Research (ASAR), Pune, Maharashtra 411007, India; 2 Department of Community Medicine, Maulana Azad Medical College, New Delhi 110002, India; 3 Pallium India, Thiruvananthapuram, Kerala 695011, India; 4 WHO Collaborating Centre for Training and Policy on Access to Pain Relief, Thiruvananthapuram, Kerala 695011, India; † Contributed equally

**Keywords:** palliative care, access to care, geographical access, health policy

## Abstract

**Background:**

Nearly 7–10 million people require palliative care (PC) in India, with less than 4% having access to it. This study aimed to assess the geographical accessibility of PC in India and estimate changes in accessibility based on its delivery from different levels of the public health system.

**Methods:**

Pallium India’s 2022 directory provided a list of active palliative care centers-Pallium India (PCC-PI). We analysed the density of PCC-PIs per ten million population, the median travel time to the nearest centre using a motorised vehicle and the access population coverage. PC delivery scenarios combining primary, secondary and tertiary public healthcare centres were created to evaluate changes in access.

**Results:**

In 2022, India had 526 PCC-PI, with a density of four per ten million population. The highest densities were in Lakshadweep, Goa and Kerala. The median (IQR) travel time to the nearest PCC-PI was 118 (71, 179) minutes, and 23.6%, 39.8% and 71% of people lived within 30, 60 and 120 minutes, respectively. Rural areas had worse access than urban areas, with considerable variation across states. States like Kerala and Chandigarh had near-universal access, while Madhya Pradesh, Odisha and Bihar had much lower coverage. Access improved significantly when PC was integrated into all levels of the healthcare system.

**Conclusion:**

Access to PC in India is limited, especially in rural areas. Expanding integration with the public health system could enhance access, ensuring more equitable care nationwide.

## Introduction

The World Health Organisation defines palliative care (PC) as ‘an approach that improves the quality of life of patients (adults and children) and their families who are facing problems associated with life-threatening illness’ [1]. Realising the impact of PC on the quality of life of patients and caregivers, in 2014, the World Health Organisation passed a resolution urging member states to provide PC services as a part of comprehensive care [2]. Four in ten people in India are estimated to have at least one chronic disease, and this burden is expected to rise [3, 4]. With the rising incidence of chronic diseases, the need for PC is also expected to rise in India to address serious health-related suffering, which is commonly associated with end-stage chronic diseases [5–7].

PC is most commonly needed for patients suffering from cardiovascular diseases, chronic respiratory diseases, cancer and neurological illnesses, among others. It is estimated that less than 4% of people in need of PC have access to it in India [8]. Specifically, in patients with last-stage cancer, who account for nearly one–third of all PC needs, the unmet need for PC is 98.3% in India [9]. There are various barriers to the poor delivery of PC in India [10]. Inequitable distribution of services, when available and poor geographic access to health centres with available services act as significant barriers to PC delivery in India.

Various national-level interventions like the National Program for PC (NPPC) [11], the National Health Policy 2017 [12] and the National Program for Prevention and Control of Non-Communicable Diseases (NPNCD) [13] guide the provision of PC from the public health system in India. However, private centres are leading the way in service delivery due to the lack of implementation of the existing programs for PC in India [14]. Moreover, as per the guidelines of the National Medical Commission, it is not mandatory to have a department for PC to establish a medical college [15]. The limited training courses and limited awareness of PC in India also make establishing PC departments in public hospitals at all service delivery levels – primary, secondary and tertiary – challenging [16].

With limited centres providing PC, it becomes important to understand the geographical distribution of these centres and geographical accessibility, i.e., the time taken to reach the nearest centre, to plan further scale-up of PC services in India [17]. Since people requiring PC are most often bed or homebound, the proximity to the centre with PC services becomes all the more crucial to understand, as poor accessibility will increase the cost of care due to the financial burden of the cost of travel and loss of wages of the caregiver [18].

Since India continues to be predominantly rural with most of the health centres concentrated in urban areas, it also becomes essential to study the differential access to PC between rural and urban areas. Even though individual-level barriers to access to PC have been studied, the geographical distribution and accessibility to PC and its urban–rural disparity in India remain unstudied [19].

We aimed to assess the multiple measures of geographic accessibility to PC centres, including density per 10 million people, travel time to the nearest PC centre and the access population coverage (APC) of centres at the national and state levels. We provide motorised accessibility estimates for 36 Indian states/union territories (UTs). We also assessed the access disparities for people living in rural and urban areas. Lastly, we also assessed how access to PC would change based on different scenarios of PC delivery from different levels of the public health system, as recommended by NPPC and NPNCD programs.

## Methodology

### Study setting

India is the seventh-largest and most populated country in the world, with a projected population of more than 1.4 billion in 2023 [20]. India has 28 states and 8 UTs. A majority of the Indian population resides in rural areas, with less than 30% of the population residing in urban areas. The landscape varies from hilly terrain in the northernmost and north-eastern part of the country, plains in the northern region, to a plateau in the southern peninsular region of the country. The terrain is important to remember while trying to understand geographical access to healthcare in the country. India has a mixed healthcare system with both public and private providers [21]. The public health system is multi-layered. Primary care in India is provided through Health and Wellness Centres (sub-centres and primary healthcare centres (PHC)), secondary care through Community Health Centres (CHCs) and tertiary care through District Hospitals (DHs) and Teaching Hospitals (THs).

For this study, both public and private THs were included under the public health system, as common guidelines of the National Medical Commission, Government of India, strictly regulate them. Since morphine cannot be prescribed from sub-centres in India, they were not included in assessing access to PC. Thus, the smallest unit of the public health infrastructure included was the primary health centre (PHC).

### Data sources

#### PC centres

A list of functioning PC centres in the country was curated using Pallium India's website directory [29]. Pallium India is India’s leading PC non-profit and advocacy organisation, which was started in 2003. The directory of PC centres was created in 2010 with the primary objective of assisting patients and medical and non-medical professionals to locate a PC centre in their vicinity. The organisation’s regional state/UT-level facilitators support the establishment of new centres and keep track of active palliative care centers-Pallium India (PCC-PI) in their respective states/UTs. The regional facilitator (RF) reaches out to the head of the centre and gets their approval for being part of the directory. After their approval, the RF collects the details about that centre's services in a standard form. In addition to the name, address and contact details of the PC centre, this form also includes information on the availability of morphine, any specialised PC and the type of services available, such as outpatient, inpatient or home care. Once this information is received from the centre, it is updated to a master database of centres maintained in an Excel sheet. The directory is updated every 6 months. For our analysis, we used the directory that was last updated in December 2022.

#### Public health centres

To comprehensively map the public health centres, the study necessitated the collection, extraction and collation of data from multiple data sources (Supplementary Table 1). The geolocation information for the Primary Health Centeres (PHCs) and CHCs was obtained from the Geographic Information System (GIS) dataset of the Pradhan Mantri Gram Sadak Yojana (PMGSY) [22]. The PMGSY is a fully centrally funded initiative by the Government of India aimed at providing year-round connectivity to previously inaccessible areas as part of a poverty reduction effort. We used the PMGSY GIS dataset from October 2021 to extract geo-coordinates and other relevant geographic details for the health facilities under examination, specifically the PHCs and CHCs. However, GIS data for PHCs were unavailable in 52 districts, and CHC data were missing for 90 districts. The location of DHs was extracted from the National Institute for Transforming India (NITI) Aayog’s Report on the evaluation of DHs published in 2021 [23]. Lastly, data on the location of THs were extracted from the National Health Profile Report 2022 published by the Central Bureau of Health Intelligence [24].

#### Access outcomes

Data on motorised friction rasters for every square kilometer were obtained from the Malaria Atlas Project 2019, an international research collaboration focusing on mapping the global response to malaria [25]. The friction rasters contain information related to the transport network in the given region and the factors that affect the time taken to move from one location to the other. Road, rail networks, navigable rivers and shipping lanes are included in the transport networks. Environmental factors, such as land cover and slope, affect the travel speed and political factors include national and state/UT boundaries that impact travel time [26].

#### Population projections and national and state-level borders

High-resolution (1 km^2^) United Nations adjusted population counts from the WorldPop dataset for 2020 for India served as our source of population data [27]. The administrative borders of India were drawn from the publicly available shapefile [28].

### Outcomes

We had three primary outcomes for the situational analysis. First, we report the density of PC centres per ten million people to understand their geographic distribution relative to the regional population. Second, we report the travel time to the nearest PC centre. Using the granular estimates of travel time for each 1 km^2^ pixel or grid cell, we report the median and interquartile range (IQR) values at aggregate group levels. Lastly, we report the APC, which we defined as the percentage of the population with timely access to the nearest PC centre. We estimated the timely access for motorised transport and considered timely access as within 30, 60 and 120 minutes. APC combined the population and timeliness aspects of access to care.

For our secondary outcome, we report the same geographical accessibility outcomes mentioned above for different models of PC service delivery through public health centres and THs (Table 1).

### Data analysis

For the geocoding of the centres, addresses were cleaned manually for improved machine readability. We used the Google Maps Application Programming Interface for geocoding. For locations that returned multiple sets of coordinates, the ones with the most relevant address string were chosen. Geo-coordinates were used to identify and remove duplicates and points extending beyond India's latitude and longitude limits. State-wise population data were extracted by imposing administrative boundaries.

For travel times, the Dijkstra algorithm was utilised to compute the minimum time required to traverse the friction surface from every pixel (grid cell) on the map to the geo-coordinates of every PC and public health centre. The algorithm was implemented for motorised transport. As walking long distances is not feasible for PC patients, we did not calculate travel time for a walking mode of transport. Lakshadweep and the Andaman & Nicobar Islands were excluded from our analysis due to methodological limitations in assessing access in regions where motorised road transport is not the primary mode of travel for healthcare access.

Access time for motorised transport was set at thresholds of 30, 60 and 120 minutes per previously published literature [30–32]. A binary accessibility raster was created with ‘1’s for pixels that fulfilled the timeliness criterion of each proxy variable and not applicable (‘NA’s) for cases otherwise. This raster was then overlaid on the population raster (extent matched). The population figures at each pixel were multiplied with the weights (i.e., ‘1’s and ‘NA’s) to get the population number with timely access.

The main analysis looked at the geographical access to PCC-PI and the secondary analysis looked at the geographical access to the centres as per the scenarios mentioned in Outcomes. Duplicates between Pallium India’s directory and the list of public health centres were removed during the analysis of the secondary outcome.

## Results

### Situational analysis of access to PCC-PI

#### PCC-PI density

The Pallium India’s directory contained 526 PCC-PI in 2022. Outpatient, inpatient and home care were provided in 410 (77.9%), 324 (61.6%) and 381 (72.4%) centres, respectively. Of the 504 centres with available data, morphine was present in 333 (66.1%) centres. At least one trained healthcare worker was present in 477 (90.7%) centres. Services were free at 371 (73%) out of 508 centres with available data and 19 (3.7%) centres provided free service to only those from poor socio-economic backgrounds. Of all centres, 44.5% centres were present in Kerala, and no centres were present in Andaman & Nicobar Islands, Dadra & Nagar Haveli, Daman & Diu and Ladakh. The PCC-PI density at the national level was four centres per ten million population. In states/UTs with PC centres, the density was the highest in Lakshadweep with 147 centres for every ten million, followed by Goa (96 centres/10 million) and Kerala (66 centres/10 million) (Table 2).

#### Timely access to PCC-PI

Nationally, the median (IQR) travel time to the nearest PC centre was 118 (71, 179) minutes. The median (IQR) time was longer for rural (120 (72, 180) minutes) than for urban areas (57 (16, 109) minutes).

Notable differences were noticed in access to the nearest PC centre at the state/UT level. The median time to reach the nearest PCC-PI was the lowest for Chandigarh (median (IQR) = 3 (2, 5) minutes) and the highest for Ladakh (median (IQR) = 591 (412, 881) minutes) (Table 3).

Twenty-nine states/UTs had a median time longer than 30 minutes. The median travel time was worse for rural areas than for urban areas in all states/UTs (Table 3). The state/UT with the lowest rural median travel time was Chandigarh (8 (6, 9) minutes) and rural Ladakh had the highest median travel time of 591 (412, 880) minutes. Similarly, the median travel time of urban Chandigarh (3 (2, 5) minutes) was the lowest and urban Ladakh (137 (137, 145) minutes) was the highest. Compared with the national median travel time, 14 states/UTs did worse.

#### APC of PCC-PI

Nationally, only 23.7% of the population resided within 30 minutes, 39.9% within 60 minutes and 71% within 120 minutes of the nearest PCC-PI. The coverage of PCC-PIs was worse in rural areas with only 11.8% of the population within 30 minutes, 29.3% within 60 minutes and 65.2% within 120 minutes as compared to the urban areas, where 55.6% of the population was within 30 minutes, 68.3% within 60 minutes and 86.2% within 120 minutes of the nearest PCC-PI.

**Figure 1.**
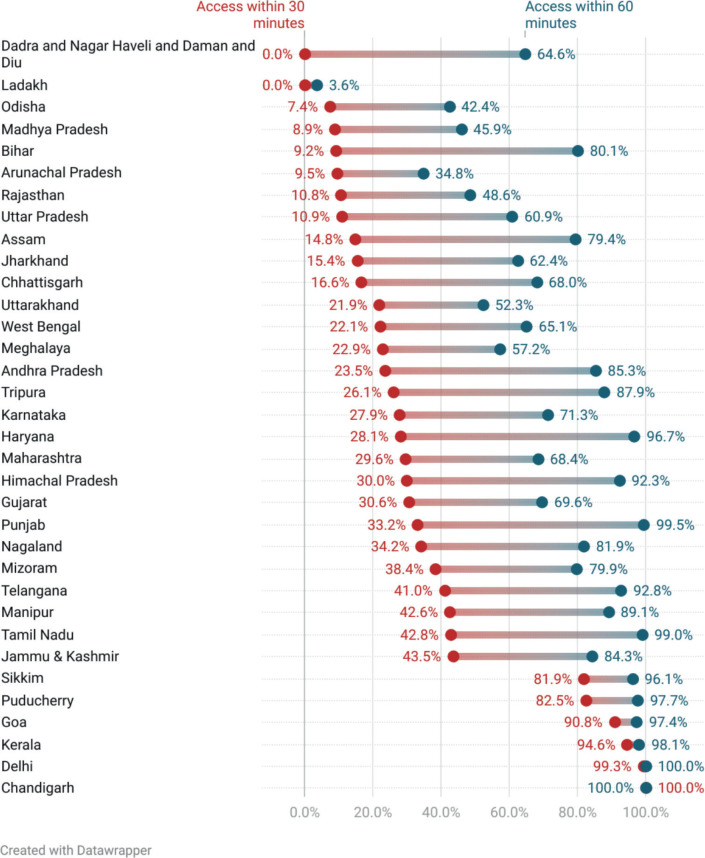
State-level APC of PCC-PI at 30-minute and 120-minute time thresholds.

**Figure 2.**
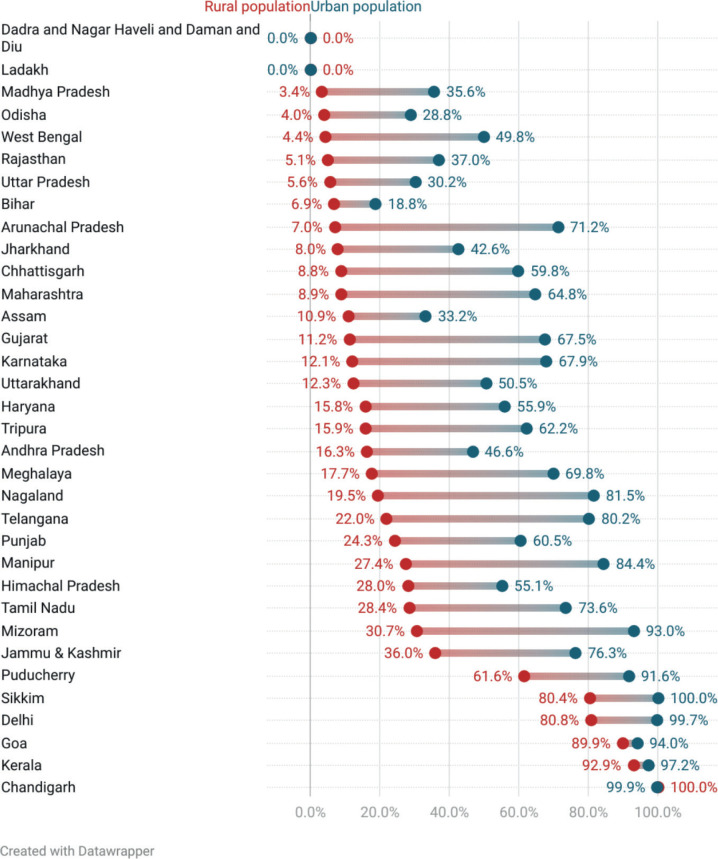
Rural and urban disparity in APC for PCC-PI at a 30-minute time threshold.

Among the states/UTs, 28, 20 and 6 states/UTs had less than 50% population within 30 minutes, 60 minutes and 120 minutes, respectively. Chandigarh, Delhi, Kerala and Goa had more than 90% of the total population within 30 minutes (Figure 1). When compared with the percentage of the population within 30 minutes at the national level, 15 states/UTs did worse. The percentage of the population with access within 30 minutes and 60 minutes was worse for rural areas as compared to urban areas in all states/UTs (Table 4). In states/UTs with at least one PC centre, Madhya Pradesh had only 3.3% of the population with access within 30 minutes as compared to Chandigarh and Kerala where 100% and 93% had access to the nearest PCC-PI within 30 minutes. Similarly, the percentage of the population with access within 30 minutes was the lowest for urban Bihar (18.8%) and the highest for urban Sikkim (100%). Rural populations of Chandigarh, Goa and Kerala had a higher percentage of the population within 30 minutes of access than urban populations of 26 states/UTs (Figure 2).

The best and worst performing states in terms of median travel times and APC are summarised in Table 5.

### Scenarios of PC delivery from PCC-PI and public health centres

#### Scenario I (PCC-PI + THs + DHs)

*Travel times:* Nationally, the median (IQR) travel time to the nearest centre using a motorised vehicle was 51 (30, 82) minutes. The travel duration was longer for rural (51 (31, 83) minutes) than for urban areas (14 (4, 32) minutes).

Among state/UTs, Chandigarh had the shortest median (IQR) travel time of 3 (2–4) minutes and Arunachal Pradesh had the longest, 381 (132, 860) minutes. Travel times for rural areas were longer than for urban areas in all states/UTs (Table 6).

*APC:* About 54.5% of the national population resided within 30 minutes, 86.4% within 60 minutes and 98.4% within 120 minutes of their nearest centre. Rural areas had lower APC than urban areas for access within 30 minutes (42.5% versus 87.2%), 60 minutes (82.0% versus 98.4%) and 120 minutes (97.8% versus 100.0%).

Among state/UTs, Delhi (100%), Chandigarh (100%), Puducherry (97.7%), Kerala (95.9%), Goa (93.5%) and Dadra & Nagar Haveli and Daman & Diu (93.1%) had more than 90% of the population within 30 minutes of the nearest centre. When compared with the percentage of the population within 30 minutes at the national level, 13 states/UTs did worse. The percentage of the population within 30 minutes was worse for rural areas than for urban areas in all states/UTs (Table 7).

#### Scenario II (Scenario II + CHCs)

*Travel times:* Nationally, the median (IQR) travel time for the total population to the nearest centre was 31 (15, 54) minutes. The travel duration was longer for rural (32 (18, 56) minutes) than for urban populations (8 (3, 19) minutes).

Among the state/UTs, Chandigarh had the shortest median (IQR) travel time of 3 (2, 4) minutes and Ladakh had the longest time of 350 (171, 634) minutes. Travel times for rural areas were longer than for urban areas in all states/UTs (Table 6).

*APC:* About 76.7% of the national population resided within 30 minutes, 95.2% within 60 minutes and 99.1 within 120 minutes from the nearest centre. Rural areas had lower APC compared with urban areas for 30 minutes (69.7% versus 95.8%), 60 minutes (93.5% versus 100%) and 120 minutes (98.7% versus 100%).

Among the state/UTs, Delhi (100%), Chandigarh (100%), Puducherry (97.7%), Haryana (97.4%), Punjab (96.4%), Kerala (96.4%), Goa (93.6%) and Dadra & Nagar Haveli and Daman & Diu (93.3%) states/UTs had APC of more than 90% for 30 minutes. When compared with the APC for 30 minutes for the national population, 17 states/UTs did worse. The APC for 30 minutes was worse for rural areas as compared to urban areas in all states/UTs, except Chandigarh, where 100% of both rural and urban populations were within 30 minutes of the nearest centre (Table 7).

#### Scenario III (Scenario II + Primary Health Centeres)

*Travel times:* Nationally, the median (IQR) travel time to the nearest centre was 16 (7, 33) minutes. The travel duration was longer for rural (16 (7, 33) minutes) than for urban areas (4 (2, 8) minutes).

Among states/UTs, Chandigarh had the shortest median (IQR) travel time of 3 (1, 4) minutes, while Ladakh had the longest time of 301 (117, 515) minutes. Travel times for rural areas were longer than for urban areas in all states/UTs (Table 6).

*APC:* About 92.8% of the national population resided within 30 minutes, 98% within 60 minutes and 99.3% within 120 minutes of their nearest centre. Rural areas had lower APC than urban areas for 30 (90.3% versus 100%), 60 (97.3% versus 100%) and 120 minutes (99.0% versus 100%).

Nineteen states/UTs had over 90% of their population within 30 minutes of the nearest centre by motorised transport (Table 7). When compared with the percentage of the population covered within a 30-minute drive at the national level, 17 states/UTs did worse.

#### Scenario IV (Scenario III without PCC-PI)

*Travel times:* Nationally, the median (IQR) travel time to the nearest centre was 16 (7, 33) minutes. The travel duration was longer for rural (16 (7, 33) minutes) than for urban areas (4 (2, 8) minutes).

Among the states/UTs, Chandigarh had the shortest travel time of 3 (2, 4) minutes and Ladakh had the longest travel time of 301 (117, 585) minutes. Travel times for rural areas were worse than for urban areas in all states/UTs (Table 6).

*APC:* Around 92.8% of the total population resided within 30 minutes, 98.0% within 60 minutes and 99.3% within 120 minutes of the nearest centre. Rural areas had lower APC than urban areas for 30 minutes (90.3% versus 100%), 60 minutes (97.3% versus 100%) and 120 minutes (99.0% versus 100%).

Among the states/UTs, 19 states/UTs had a population of more than 90% within 30 minutes of the nearest centre (Table 7). When compared with the APC of the national population for 30 minutes, 18 states/UTs did worse.

**Figure 3.**
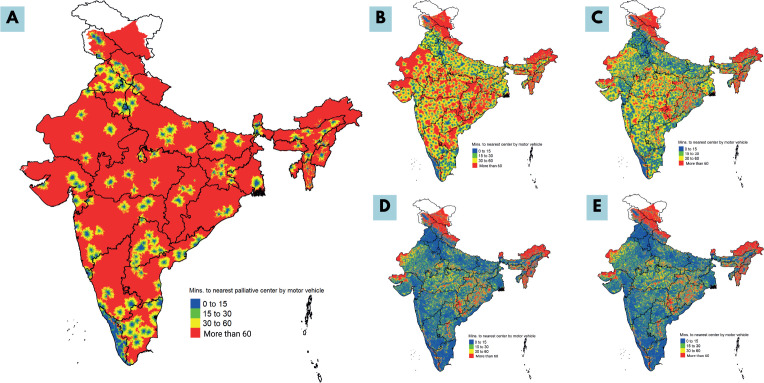
Heatmap showing timely access to PCC-PI (a) PCC-PI (b) Scenario I (c) Scenario II (d) Scenario III and (e) Scenario IV with the colors ‘Blue’, ‘Green’, ‘Yellow’ and ‘Red’ highlighting access within 15 minutes, 15–30 minutes, 30–60 minutes and more than 60 mintues respectively.

**Figure 4.**
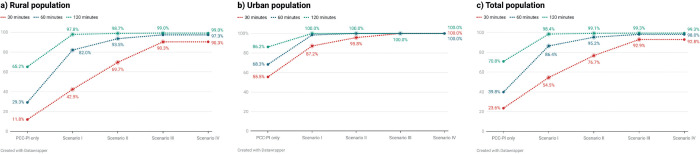
Improvement in APC in (a) rural (b) urban and (c) total population across different scenarios of access.

A comparison of all four scenarios revealed a progressive improvement in access in terms of time to reach the nearest centre and the APC at all time thresholds, i.e., 30, 60 and 120 minutes (Tables 7–9). Figure 3 shows heatmaps of access to PCC-PI and centres in Scenarios I–IV. The progressive reduction in area shaded ‘Red’ (highlighting region in which time taken to nearest centre would be more than one hour, i.e., APC of more than 60 minutes) visually highlights the improvement in access. At the national level, population estimates in the red zone reduced from 827,498,048 (~827 million people) in the situational analysis to 187,356,672 (~187 million), 65,889,280 (~66 million), 26,909,952 (~27 million) and 26,937,344 (~27 million) in Scenarios I–IV, respectively. When compared to the APC of PCC-PI, the APCs from scenarios I to IV were progressively improved (Figure 4). The travel times and APC of scenario III were the same or nearly similar to scenario IV, highlighting that the public health system alone could provide adequate access to PC in India.

## Discussion

India has only four PC centres per 10 million people. The median time to reach the nearest PC centre was nearly 2 hours, and only 23.7% of people had access to PC services within 30 minutes of motorised access. However, the coverage varies in different regions of the country. Kerala, a state in southern India, with 2.5% of the country's population, performed better than states with similar populations, like Punjab and Assam. This is because 44.5% of all the PC centres in the country were present in Kerala. This has resulted in 94.6% of the population in Kerala being within 30 minutes of the nearest PC centre, with a median (IQR) travel time to the nearest centre of 14 (8, 28) minutes. Contrary to this, the state of Uttar Pradesh, with nearly 17% of the country’s population, had only 12 (2.3%) PC centres with 10.9% of the population within 30 minutes of the nearest PC centre and a median (IQR) travel time to the nearest centre of 111 (74, 152) minutes.

Better access in the UTs of Chandigarh and Delhi and the state of Goa compared to the rest of the states/UTs can be explained by the relatively smaller size of the territories, a small population and greater urbanisation. Arunachal Pradesh, Manipur and Sikkim, states in northeast India, despite having a PC centre density better than the national average, had worse accessibility in terms of median time taken to reach the nearest centre. This can be explained by the hilly terrain of the northeastern region of the country, which would have increased the travel time to the centre.

We also noted a stark urban-rural disparity in access to PC centres. The median travel time ranged from 1 to 591 minutes in rural areas compared to 3–137 minutes in urban areas. Similarly, in states/UTs with PC centres, the APC within 30 minutes of the nearest centre ranged from 3.3% to 100% in rural areas, as compared to 18.8% to 100% in urban areas.

This can be explained by the poorer road infrastructure in rural areas, which increases travel times. In 2018–2019, the average road density in urban and rural areas was 5296.3 and 1458.1 per 1,000 km^2^, respectively [33]. An urban-rural disparity in the establishment of health centres has also contributed to the difference in travel times in urban and rural areas. As health professionals in India prefer to practice in urban areas, owing to the availability of better amenities and more opportunities for career growth, more centres tend to be established in urban areas [34].

In the scenarios incorporating PC at different levels of the health system, the access was noticed to be best in the third scenario, which included all the public health centres and the PCC-PI as per Pallium India’s directory. However, the access parameters were only marginally better than the scenario in which only the public health centres were included. This highlights that the public health system can effectively deliver PC in India with a supplementary role from private centres in rural and remote parts of the country where access remains poor despite complete engagement of the public health system.

A similar analysis has been attempted to understand access to PC services in high-income countries. The accessibility reported in these regions in terms of APC is significantly better than the Indian scenario. In Switzerland, Germany, Ireland, Spain and Switzerland, 95%, 86%, 84% and 79%, of the population, respectively, were reported to live within 30 minutes from the nearest centre with specialised PC services [35, 36].

While Chandigarh (100%), Delhi (99.2%), Kerala (95.7%) and Goa (92.8%) reported better access in terms of APC compared to Germany (86%), all other states/UTs in India reported access poorer than Ireland (84%). Similar to the urban-rural disparity in India, the access to PC, in terms of travel times, in rural areas of Virginia Tennessee and West Virginia in the United States of America was found to be nearly five times that the travel times in the urban areas of the respective states [37].

In this work, we have used multiple measures of geographic accessibility. Density, a commonly used metric, provides information on the geographical distribution of facilities by population. However, it fails to provide information on timely access to facilities. The presence of a road network, its quality, traffic and the availability of public or personal transport are some of the factors that impact accessibility and can be influenced by policymakers.

Our study is the first to calculate travel times and APC for PC centres in India. We preferred travel times over distances as pragmatically travel times better incorporate various infrastructural and geographical barriers mentioned previously. However, travel times fail to convey the population falling in the catchment areas of the centres. By combining time with the percentage of the population living around the centres, APC uses the percentage of the population that can access care in a given period. Therefore, APC gives us a more complete picture of the geographical accessibility to care in a region. We recommend that APC be used to guide policy regarding the establishment of future health centres in the country.

There is a need to address the disparities in geographical accessibility to PC centres through a strategic placement of new centres. This can be done by the use of location-allocation models. Using these models, policymakers can improve accessibility to centres by opening new centres at optimised locations [38]. Since patients receiving PC are home or bedbound, our analysis also shows that the existing centre-based approach to PC may not be able to universalise its access.

Therefore, policymakers need to emphasise the home-based model of PC service delivery. Through various scenarios, we also highlight how access to PC can be improved using existing public health infrastructure. Once the existing public health infrastructure is equipped with PC services, access to the ones in rural areas can be improved by improving road networks through schemes such as the PMGSY. As proposed by the NPPC, community health workers, i.e., Accredited Social Health Activists could be crucial in identifying patients requiring PC in the community and referring them to the nearest PHC or organising a visit to the patient’s house by informing the medical officer of the nearest PHC. Areas that face poor access to the nearest centre could be accessed through mobile clinics by strategically identifying nearest public health infrastructure with available services. Through these efforts, it will be possible to universalise access to PC services in India.

### Limitations and strengths

The study has multiple limitations. First, this study relied only on Pallium India’s directory of PC centres and state governments or the Indian Association of PC (IAPC) were not approached to cross-check the completeness of the list. Therefore, it is possible that Pallium India’s directory missed smaller or less well-known PC facilities. This might have resulted in an underestimation of PC access. However, prior to using the directory the completeness of the directory was verified by the RFs at Pallium India to ensure the list was up to date and only active centres were included in the list. The list was also cross-checked with the directory maintained by the IAPC and Pallium India’s directory was found be larger and more up to date making it the largest available list of active centres available in India. This limitation of our study also highlights the need for state-maintained list of PC centres to ensure that not just researchers but also the public is aware of the available PC centres in their region. We have addressed this limitation by reporting changes in PC accessibility through different scenarios considering the delivery of services from different levels of the public health system. Second, our analysis inherited the assumptions and limitations of all the parent/source datasets. Third, to calculate the APC estimates, we did not take into account the access to or ownership of motor vehicles. Only 21% of Indian households owned two-wheelers, and 4.7% owned cars, jeeps or vans as per the 2011 census [39]. Although India meets WHO’s norm of 1 ambulance per 100,000 people, there are large disparities in the availability of ambulance services among states/UTs [40]. Fourth, health centre-related factors like affordability of care, functional timings, quality of services provided at the centres and the capacity of the centres to provide care were not considered while assessing accessibility.

Despite these limitations, our study has several strengths. This is the first attempt to understand access to PC in a lower-middle-income country. Considering the huge burden of non-communicable diseases in India, it becomes essential to understand the access to PC. The major strength of our study is that accessibility has been defined using three outcome measurements - PC centre density, time to reach the nearest centre and APC within multiple time frames. Not only have we reported a state-level analysis on access to PC but also done an urban-rural comparison. This will help policymakers in deciding not only how many more centres are needed in each state but also in identifying the exact locations where building a centre would improve geographic accessibility.

## Conclusion

Comprehensive tools like median travel times and APC can be used to study accessibility to healthcare services. Our study found that Chandigarh, Delhi, Kerala and Goa had good access to PC while most other states/UTs, especially in the north and northeastern parts of the country need to improve accessibility to PC in the region. We also found a significant urban-rural disparity in access to PC. Future research should assess access to specific PC services like morphine availability among others and also assess accessibility for different demographic groups along with its impact on quality of life and disease outcomes. There is also a need to study access to home-based PC services either through mobile clinics or through community engagement and involvement of community healthcare workers.

## List of abbreviations

APC, Access population coverage; CHC, Community Health Centres; DH, District Hospitals; GIS – Geographic Information System; IQR, Inter Quartile Range; NPNCD – National Program for Prevention and Control of Non-Communicable Diseases; NPPC – National Program for Palliative Care; PCC-PI – Active palliative care centres; PHC, Primary Health Centres; PMGSY - Pradhan Mantri Gram Sadak Yojana; RF, Regional Facilitator; TH, Teaching Hospitals; UT, Union Territories.

## Conflicts of interest

The author(s) declare that they have no conflicts of interest.

## Funding

This study did not receive any funding.

## Data sharing statement

Data will be made available upon reasonable request to the corresponding author.

## Figures and Tables

**Table 1.** Scenarios of PC service delivery by including different levels of the public health system.

Scenario	Service delivery centres	Description
I	PCC-PI, THs and district hospitals (DH)	Delivery of PC services from TH and DH has been proposed by the NPNCD. This scenario there includes PCC-PI as per Pallium India’s directory along with TH and DH in the public health system.
II	Scenario I + CHCs	Delivery of PC from the tertiary (TH) and secondary hospitals (DH and CHC) in the public health system along with PCC-PI as per Pallium India’s directory.
III	Scenario II + PHCs	Delivery of PC from all levels of the public health system has been proposed by the NPPC and Ayushman Bharat program. Therefore this scenario includes PCC-PI as per Pallium India’s directory and all levels of service delivery in the public health system (TH, DH, CHC and PHC).
IV	Scenario III - PCC-PI	This scenario excludes the PCC-PI as per Pallium India’s directory, which are predominantly private health centres, to understand the access to PC if only the public health system delivered the services.

**Table 2.** PC centres per 10 million population in India.

State/UT	Projected population (2022)	Number of PC centres (PCC-PI)	PCC-PI per 10 million people
Andaman & Nicobar Islands	402,000	0	0.0
Andhra Pradesh	90,879,000	13	1.4
Arunachal Pradesh	1,548,000	1	6.5
Assam	35,378,000	12	3.4
Bihar	124,919,000	8	0.6
Chandigarh	1,219,000	5	41.0
Chhattisgarh	29,836,000	7	2.3
Dadra & Nagar Haveli and Daman & Diu	1,170,000	0	0.0
Delhi	20,965,000	15	7.2
Goa	1,567,000	15	95.7
Gujarat	70,648,000	14	2.0
Haryana	29,846,000	6	2.0
Himachal Pradesh	7,431,000	7	9.4
Jammu & Kashmir	13,804,000	6	4.3
Jharkhand	38,969,000	6	1.5
Karnataka	67,268,000	12	1.8
Kerala	35,633,000	234	65.7
Lakshadweep	68,000	1	147.1
Madhya Pradesh	85,548,000	9	1.1
Maharashtra	125,411,000	22	1.8
Manipur	3,194,000	3	9.4
Meghalaya	3,318,000	1	3.0
Mizoram	1,227,000	7	57.0
Nagaland	2,213,000	3	13.6
Odisha	44,162,000	6	1.4
Puducherry	1,608,000	4	24.9
Punjab	30,535,000	8	2.6
Rajasthan	80,153,000	9	1.1
Sikkim	683,000	2	29.3
Tamil Nadu	76,631,000	44	5.7
Telangana	37,907,000	18	4.7
Tripura	41,09,000	1	2.4
Uttar Pradesh	233,297,000	12	0.5
Uttarakhand	11,518,000	5	4.3
West Bengal	98,604,000	10	1.0

**Table 3.** State-wise time to reach the nearest PC centre (PCC-PI) for total, rural and urban populations.

S. No.	State/UT name	Time to reach the nearest centre (minutes)					
		Total population		Rural population		Urban population	
**Mean (SD)**	**Median (IQR)**	**Mean (SD)**	**Median (IQR)**	**Mean (SD)**	**Median (IQR)**
1	Arunachal Pradesh	715.4 (613.0)	505.1 (247.9, 1011.7)	713.9 (611.2)	504.5 (247.8, 1009.4)	67.3 (71.9)	34.3 (5.1, 146.6)
2	Assam	115.4 (100.5)	90.6 (58.2, 142.6)	117.1 (101.7)	92.0 (59.4, 143.9)	77.2 (59.0)	65.3 (34.7, 97.8)
3	Chandigarh	3.8 (2.3)	3.2 (1.9, 5.0)	7.9 (2.1)	8.4 (6.1, 9.3)	3.5 (2.0)	3.1 (1.8, 4.9)
4	Karnataka	113.5 (56.6)	108.3 (68.8, 156.6)	114.5 (56.2)	109.2 (69.7, 157.3)	69.5 (57.9)	61.3 (11.9, 112.2)
5	Manipur	202.0 (203.6)	128.0 (69.4, 270.8)	204.8 (203.4)	130.7 (71.6, 274.0)	22.3 (19.3)	17.7 (6.5, 32.7)
6	Meghalaya	153.9 (107.2)	139.0 (77.3, 207.3)	154.5 (107.3)	139.3 (77.8, 207.6)	87.0 (78.3)	57.7 (13.8, 170.2)
7	Mizoram	142.4 (108.7)	110.1 (60.7, 198.1)	142.4 (108.5)	110.2 (60.9, 198.1)	12.8 (16.7)	3.7 (1.9, 36.4)
8	Nagaland	129.2 (88.8)	106.8 (72.2, 161.8)	129.8 (88.3)	107.2 (73.1, 162.4)	26.2 (33.7)	5.2 (3.3, 52.8)
9	Punjab	52.4 (23.5)	51.9 (34.6, 69.6)	53.2 (23.0)	52.7 (35.4, 70.1)	33.9 (25.9)	33.0 (9.4, 50.9)
10	Rajasthan	158.4 (87.7)	143.5 (102.1, 193.9)	159.1 (87.6)	144.0 (102.6, 194.4)	101.5 (64.2)	107.0 (52.4, 142.5)
11	Sikkim	253.8 (262.0)	169.0 (44.2, 383.5)	254.1 (260.5)	169.8 (45.2, 384.2)	8.7 (8.0)	5.6 (2.3, 18.5)
12	Tripura	94.7 (51.8)	90.8 (60.4, 118.7)	96.2 (51.0)	91.8 (62.6, 119.8)	53.3 (39.2)	49.9 (10.9, 88.7)
13	Uttarakhand	330.7 (365.1)	205.8 (109.6, 361.0)	334.3 (366.5)	207.9 (111.8, 367.6)	95.0 (88.4)	58.3 (16.9, 171.2)
14	Telangana	78.9 (47.7)	69.5 (43.7, 106.8)	79.9 (47.4)	70.2 (44.6, 107.6)	33.9 (36.2)	13.1 (5.9, 55.0)
15	Bihar	99.0 (45.7)	96.3 (66.4, 127.3)	101.0 (46.1)	98.4 (68.1, 129.5)	80.8 (37.4)	82.0 (53.8, 105.7)
16	Kerala	39.3 (77.6)	14.4 (7.8, 28.4)	44.7 (82.9)	16.4 (9.1, 34.1)	8.6 (9.9)	6.7 (3.4, 11.5)
17	Madhya Pradesh	139.0 (57.9)	136.1 (98.6, 175.2)	139.5 (57.7)	136.5 (99.0, 175.5)	101.2 (60.9)	107.7 (56.4, 148.7)
19	Gujarat	171.3 (147.5)	133.1 (80.2, 212.9)	145.6 (95.3)	126.2 (77.5, 192.2)	75.7 (66.9)	63.8 (16.2, 115.4)
21	Odisha	174.1 (73.0)	176.0 (127.0, 219.0)	175.3 (72.6)	177.0 (128.6, 219.7)	101.0 (60.9)	98.9 (52.9, 153.0)
22	Dadra and Nagar Haveli and Daman and Diu	128.1 (31.6)	122.4 (115.7, 129.7)	128.9 (27.7)	123.9 (118.2, 131.5)	115.2 (27.7)	113.0 (99.0, 118.6)
23	Ladakh	699.9 (415.2)	591.2 (412.3, 880.6)	699.8 (415.2)	591.1 (412.2, 880.2)	144.5 (12.5)	137.4 (137.2, 144.7)
24	Jammu & Kashmir	306.9 (461.6)	130.1 (57.0, 333.6)	315.2 (466.0)	137.2 (61.7, 342.0)	32.3 (44.1)	20.2 (7.7, 35.4)
25	Chhattisgarh	163.2 (89.8)	151.3 (93.1, 222.6)	164.4 (89.4)	152.4 (94.5, 223.5)	69.0 (63.6)	45.1 (23.2, 93.6)
26	Delhi	11.2 (8.2)	8.7 (4.8, 15.9)	23.7 (5.5)	23.1 (19.7, 27.2)	9.1 (6.5)	7.5 (4.3, 12.1)
27	Goa	35.5 (39.2)	24.3 (12.7, 41.4)	37.6 (39.8)	25.8 (14.6, 43.1)	7.2 (6.6)	4.6 (2.1, 11.4)
28	Haryana	70.1 (33.1)	68.3 (43.6, 94.1)	71.5 (32.7)	69.6 (45.2, 95.1)	44.0 (30.5)	36.9 (17.8, 69.3)
29	Himachal Pradesh	342.3 (384.9)	182.2 (55.3, 526.0)	344.3 (385.0)	185.4 (56.0, 530.2)	34.2 (22.8)	33.5 (13.3, 48.5)
30	Jharkhand	120.4 (64.1)	114.4 (66.7, 168.7)	121.6 (63.6)	115.8 (68.4, 169.5)	81.0 (66.9)	55.4 (32.5, 123.3)
31	Tamil Nadu	58.8 (36.4)	54.6 (33.9, 77.8)	60.0 (36.3)	55.8 (35.2, 78.6)	32.0 (27.9)	25.2 (6.9, 49.6)
32	Uttar Pradesh	114.8 (54.3)	111.0 (73.9, 152.3)	115.9 (53.9)	111.8 (75.2, 153.0)	94.8 (57.9)	94.7 (44.5, 137.1)
33	West Bengal	124.8 (108.0)	115.0 (70.4, 155.5)	128.8 (105.2)	119.1 (77.4, 157.7)	81.8 (60.8)	66.3 (31.5, 122.5)
34	Andhra Pradesh	98.2 (51.6)	92.8 (59.4, 131.0)	99.2 (51.3)	93.6 (60.5, 131.7)	52.2 (40.6)	44.4 (17.8, 79.5)
35	Puducherry	34.5 (36.4)	11.9 (5.6, 84.0)	41.4 (37.4)	16.8 (8.4, 85.7)	20.0 (31.2)	5.0 (2.6, 11.5)
36	Maharashtra	114.9 (58.2)	108.8 (71.8, 154.0)	116.0 (57.8)	109.5 (73.0, 154.7)	70.2 (58.0)	53.1 (15.5, 119.2)

**Table 4.** APC of PCC-PI for total, rural and urban populations.

S. No	State/UT name	Access population coverage (%)								
		Total population			Rural population			Urban population		
**Within 30 minutes**	**Within 60 minutes**	**Within 120 minutes**	**Within 30 minutes**	**Within 60 minutes**	**Within 120 minutes**	**Within 30 minutes**	**Within 60 minutes**	**Within 120 minutes**
1	Arunachal Pradesh	9.5	14.5	34.8	7.0	11.7	32.6	71.2	81.7	87.7
2	Assam	14.8	38.0	79.4	10.9	34.4	78.4	33.2	55.2	84.2
3	Chandigarh	100.0	100.0	100.0	100.0	100.0	100.0	99.9	99.9	99.9
4	Karnataka	27.9	41.3	71.3	12.1	28.4	64.3	67.9	74.0	89.0
5	Manipur	42.6	71.3	89.1	27.4	61.0	84.9	84.4	99.6	100.0
6	Meghalaya	22.9	36.0	57.2	17.7	31.6	54.7	69.8	76.1	79.5
7	Mizoram	38.4	59.8	79.9	30.7	53.9	76.8	93.0	100.0	100.0
8	Nagaland	34.2	50.6	81.9	19.5	37.0	75.7	81.5	94.7	100.0
9	Punjab	33.2	72.4	99.5	24.3	66.7	99.1	60.5	89.9	100.0
10	Rajasthan	10.8	18.8	48.6	5.1	13.5	44.8	37.0	43.3	65.8
11	Sikkim	81.9	92.8	96.1	80.4	92.2	95.7	100.0	100.0	100.0
12	Tripura	26.1	47.0	87.9	15.9	38.0	85.5	62.2	79.0	96.8
13	Uttarakhand	21.9	40.8	52.3	12.3	32.8	47.5	50.5	64.6	66.6
14	Telangana	41.0	66.4	92.8	22.0	55.3	90.5	80.2	89.5	97.7
15	Bihar	9.2	28.8	80.1	6.9	26.3	78.0	18.8	39.6	89.1
16	Kerala	94.6	97.4	98.1	92.9	97.3	98.6	97.2	98.2	98.2
17	Madhya Pradesh	8.9	15.8	45.9	3.4	10.0	41.3	35.6	43.4	67.8
18	Gujarat	30.6	42.6	69.6	11.2	27.0	60.0	67.5	72.7	88.9
19	Odisha	7.4	17.4	42.4	4.0	13.9	39.1	28.8	39.4	63.3
20	Dadra and Nagar Haveli and Daman and Diu	0.0	0.0	64.6	0.0	0.0	41.6	0.0	0.0	89.2
21	Ladakh	0.0	0.0	3.6	0.0	0.0	3.6	0.0	0.0	0.0
22	Jammu & Kashmir	43.5	65.3	84.3	36.0	59.1	81.1	76.3	92.2	98.6
23	Chhattisgarh	16.6	34.7	68.0	8.8	26.6	63.1	59.8	79.5	95.4
24	Delhi	99.3	100.0	100.0	80.8	96.2	96.2	99.7	100.0	100.0
25	Goa	90.8	96.9	97.4	89.9	98.5	99.1	94.0	94.0	94.0
26	Haryana	28.1	56.5	96.7	15.8	47.7	95.0	55.9	76.4	100.0
27	Himachal Pradesh	30.0	71.7	92.3	28.0	70.1	91.6	55.1	92.6	100.0
28	Jharkhand	15.4	36.1	62.4	8.0	25.7	55.4	42.6	74.1	87.9
29	Tamil Nadu	42.8	73.5	99.0	28.4	65.7	98.5	73.6	90.3	100.0
30	Uttar Pradesh	10.9	24.2	60.9	5.6	18.3	57.8	30.2	45.5	72.0
31	West Bengal	22.1	36.3	65.1	4.4	18.1	52.2	49.8	64.6	85.2
32	Andhra Pradesh	23.5	49.1	85.3	16.3	42.3	82.4	46.6	71.0	95.1
33	Puducherry	82.5	83.1	97.7	61.6	63.3	96.1	91.6	91.8	99.0
34	Maharashtra	29.6	42.8	68.4	8.9	24.0	59.7	64.8	74.8	83.3

**Table 5.** Best and worst performing states in terms of median travel times and APC.

	Best performing	Worst performing
Median travel time		
• Total population	Chandigarh (median (IQR) = 3 (2, 5) minutes)	Ladakh (median (IQR) = 591 (412, 881) minutes)
• Urban population	Chandigarh (median (IQR) = 3 (2, 5) minutes)	Ladakh (median (IQR) = 137 (137, 145) minutes)
• Rural population	Chandigarh (median (IQR) = 8 (6, 9) minutes)	Ladakh (median (IQR) = 591 (412, 880) minutes)
• APC (Total population)		
• 30 minutes	Chandigarh (100%)	Ladakh, Dadra and Nagar Haveli and Daman and Diu (0%)
• 60 minutes	Chandigarh (100%)	Ladakh, Dadra and Nagar Haveli and Daman and Diu (0%)
• 120 minutes	Chandigarh (100%)	Ladakh (3.6%)
• APC (Urban population)		
• 30 minutes	Chandigarh, Sikkim (100%)	Ladakh, Dadra and Nagar Haveli and Daman and Diu (0%)
• 60 minutes	Chandigarh, Delhi, Sikkim, Mizoram (100%)	Ladakh, Dadra and Nagar Haveli and Daman and Diu (0%)
• 120 minutes	Chandigarh, Sikkim, Delhi, Mizoram, Manipur, Nagaland, Himachal Pradesh, Tamil Nadu, Punjab, Haryana (100%)	Ladakh (0%)
• APC (Rural population)		
• 30 minutes	Chandigarh (100%)	Ladakh, Dadra and Nagar Haveli and Daman and Diu (0%)
• 60 minutes	Chandigarh (100%)	Ladakh, Dadra and Nagar Haveli and Daman and Diu (0%)
• 120 minutes	Chandigarh (100%)	Ladakh (3.6%)

**Table 6.** State-wise median travel times for each access scenario for total, rural and urban populations.

State/UT name	Scenario I			Scenario II			Scenario III			Scenario IV		
	Total	Rural	Urban	Total	Rural	Urban	Total	Rural	Urban	Total	Rural	Urban
Arunachal Pradesh	381.1 (132.0, 860.4)	380.4 (131.9, 858.3)	4.9 (1.1, 15.4)	339.6 (111.6, 747.5)	339.0 (111.5, 745.5)	1.9 (1.0, 6.7)	297.7 (91.4, 651.9)	297.2 (91.4, 650.3)	1.4 (0.9, 2.5)	297.7 (91.4, 651.9)	297.2 (91.4, 650.3)	1.4 (0.9, 2.5)
Assam	46.0 (26.7, 81.4)	47.8 (27.9, 83.8)	20.7 (8.0, 32.1)	30.1 (16.0, 62.4)	31.4 (16.8, 64.7)	12.4 (5.1, 22.2)	16.4 (7.0, 44.1)	17.4 (7.6, 46.4)	4.5 (2.3, 9.0)	16.4 (7.1, 44.1)	17.4 (7.6, 46.4)	4.7 (2.4, 9.1)
Chandigarh	2.5 (1.6, 3.6)	5.9 (4.8, 7.0)	2.4 (1.4, 3.5)	2.5 (1.6, 3.6)	5.9 (4.8, 7.0)	2.4 (1.4, 3.5)	2.5 (1.6, 3.6)	5.7 (4.8, 7.0)	2.4 (1.4, 3.3)	4.3 (3.0, 5.6)	5.9 (5.1, 7.1)	4.2 (3.0, 5.5)
Karnataka	45.0 (28.8, 64.2)	45.4 (29.4, 64.6)	8.9 (3.0, 34.5)	25.0 (15.1, 36.6)	25.3 (15.5, 36.9)	6.1 (2.6, 17.5)	8.2 (4.0, 16.8)	8.4 (4.1, 17.0)	3.3 (1.8, 5.7)	8.2 (4.0, 16.8)	8.4 (4.1, 17.0)	3.4 (1.8, 5.8)
Manipur	92.6 (37.6, 234.0)	95.0 (39.7, 236.5)	5.2 (2.9, 9.5)	85.1 (31.1, 223.6)	87.8 (32.9, 226.9)	4.2 (2.4, 5.9)	66.0 (16.4, 189.0)	68.8 (18.2, 191.6)	1.8 (1.1, 3.1)	66.0 (16.4, 189.0)	68.8 (18.2, 191.6)	1.8 (1.1, 3.1)
Meghalaya	58.1 (36.7, 93.9)	58.4 (37.1, 94.4)	8.5 (2.5, 36.5)	40.3 (21.8, 72.7)	40.6 (22.1, 73.2)	5.0 (2.0, 14.4)	27.8 (12.8, 57.8)	28.1 (13.1, 58.3)	2.9 (1.4, 6.1)	27.8 (12.9, 57.8)	28.1 (13.1, 58.4)	2.9 (1.4, 6.6)
Mizoram	97.8 (49.6, 186.2)	97.8 (49.7, 186.2)	1.8 (1.1, 2.8)	93.1 (44.3, 181.5)	93.3 (44.5, 181.5)	1.6 (1.0, 2.3)	75.3 (30.54, 164.23)	75.5 (30.6, 164.)	1.6 (1.0, 2.3)	75.3 (30.5, 164.3)	75.6 (30.7, 164.3)	1.8 (1.2, 3.0)
Nagaland	57.7 (29.6, 114.4)	58.1 (29.9, 114.9)	2.8 (1.4, 5.0)	44.9 (17.5, 100.8)	45.3 (18.0, 101.4)	2.5 (1.3, 4.2)	36.94 (9.98, 88.89)	37.3 (10.3, 89.6)	1.9 (1.1, 3.4)	36.9 (10.0, 88.8)	37.3 (10.3, 89.6)	2.5 (1.1, 4.1)
Punjab	27.1 (17.8, 36.5)	27.5 (18.6, 36.9)	7.6 (3.6, 20.5)	14.1 (8.8, 20.4)	14.4 (9.2, 20.7)	5.6 (3.0, 11.2)	6.26 (3.62, 10.25)	6.4 (3.6, 10.4)	3.6 (2.1, 5.7)	6.2 (3.6, 10.2)	6.4 (3.6, 10.4)	3.7 (2.2, 5.7)
Rajasthan	66.7 (44.2, 99.8)	67.2 (44.7, 100.3)	24.8 (5.3, 48.1)	29.9 (18.0, 47.7)	30.2 (18.3, 48.1)	7.6 (2.7, 16.4)	17.12 (8.89, 29.33)	17.3 (9.1, 29.5)	4.5 (2.0, 8.9)	17.1 (8.8, 29.3)	17.31 (9.1, 29.5)	4.7 (2.0, 9.0)
Sikkim	150.6 (34.8, 363.4)	151.3 (35.7, 363.7)	3.0 (1.0, 4.9)	149.9 (33.2, 363.3)	150.5 (34.4, 363.7)	2.3 (1.0, 4.6)	128.62 (19.44, 340.7)	129.2 (21.1, 341.1)	1.5 (0.9, 2.5)	128.7 (19.4, 340.7)	129.2 (21.1, 341.1)	2.3 (1.1, 2.9)
Tripura	39.3 (22.4, 67.1)	40.5 (23.7, 68.2)	9.7 (4.4, 22.2)	25.3 (12.9, 48.6)	26.3 (13.8, 49.9)	6.0 (3.2, 12.3)	15.08 (7.2, 34.02)	15.8 (7.6, 34.9)	4.3 (2.2, 8.3)	15.0 (7.2, 3.0)	15.8 (7.6, 34.9)	4.3 (2.2, 8.3)
Uttarakhand	64.0 (32.3, 247.4)	65.4 (33.1, 254.6)	11.9 (4.3, 30.9)	41.0 (15.6, 214.4)	42.3 (16.2, 221.9)	5.7 (2.7, 11.8)	30.33 (6.67, 175.53)	31.5 (7.0, 181.3)	3.1 (1.7, 4.9)	30.3 (6.6, 175.5)	31.5 (7.0, 181.3)	3.1 (1.7, 4.9)
Telangana	48.4 (30.6, 71.2)	49 (31.5, 71.8)	6.7 (3.2, 26.5)	31.8 (19.1, 50.5)	32.3 (19.7, 51.0)	5.6 (2.7, 14.2)	13.69 (6.98, 23.05)	13.9 (7.2, 23.3)	3.7 (1.9, 6.5)	13.7 (7.0, 23.0)	14.0 (7.2, 23.3)	4.0 (2.0, 6.7)
Bihar	34.3 (22.4, 48.7)	35.2 (23.3, 49.8)	26.3 (14.2, 38.3)	21.3 (12.5, 33.1)	22.2 (13.3, 34.3)	13.6 (5.8, 22.3)	11.19 (5, 19.93)	12.0 (5.5, 20.7)	4.9 (2.2, 11.1)	11.1 (5.0, 19.9)	12.0 (5.5, 20.7)	4.9 (2.2, 11.1)
Kerala	12.5 (6.8, 26.0)	14.4 (8.1, 32.2)	5.4 (2.9, 9.3)	8.7 (4.8, 20.2)	10.1 (5.6, 26.1)	4.3 (2.4, 7.0)	4.92 (2.77, 11.52)	5.5 (3.2, 16.3)	2.9 (1.6, 4.4)	5.4 (3.0, 12.1)	6.0 (3.3, 16.5)	3.5 (1.9, 5.2)
Madhya Pradesh	55.1 (37.4, 75.9)	55.4 (37.8, 76.1)	18.5 (3.5, 41.9)	38.8 (24.3, 56.3)	39.1 (24.6, 56.6)	9.0 (2.8, 24.8)	19.79 (11, 32.46)	20.0 (11.2, 32.6)	4.7 (2.1, 10.4)	19.8 (11.0, 32.5)	20.0 (11.2, 32.7)	4.9 (2.2, 10.5)
Gujarat	50.8 (31.8, 81.9)	48.4 (31.1, 71.9)	13.7 (4.2, 30.2)	29.6 (16.9, 49.2)	27.9 (16.4, 43.7)	7.8 (3.2, 16.8)	14.45 (6.75, 27.34)	13.4 (6.5, 23.0)	4.3 (2.2, 7.2)	14.4 (6.7, 27.3)	13.4 (6.5, 23.0)	4.4 (2.3, 7.3)
Odisha	62.1 (38.3, 95.9)	62.7 (39.0, 96.7)	20.1 (4.1, 39.9)	31.6 (16.8, 60.8)	32.2 (17.2, 61.5)	5.7 (2.4, 13.8)	21.69 (10.59, 47.44)	22.1 (11.0, 48.0)	3.8 (1.8, 8.4)	21.6 (10.5, 47.4)	22.1 (11.0, 48.0)	3.8 (1.8, 8.4)
Dadra and Nagar Haveli and Daman and Diu	19.6 (10.2, 27.4)	21.3 (13.0, 30.1)	6.9 (3.2, 18.9)	16.2 (9.8, 26.3)	18.1 (11.9, 29.2)	6.7 (3.2, 14.5)	8.42 (4.68, 12.72)	9.7 (6.1, 13.5)	3.1 (2.1, 5.2)	8.4 (4.6, 12.7)	9.7 (6.1, 13.5)	3.1 (2.1, 5.2)
Ladakh	367.1 (185.6, 656.3)	367.0 (185.5, 655.8)	0.6 (0.5, 8.1)	349.9 (170.6, 633.8)	349.8 (170.5, 633.4)	0.6 (0.5, 8.1)	300.96 (117.35, 585.3)	300.9 (117.2, 584.9)	0.6 (0.5, 8.1)	300.9 (117.3, 585.3)	300.9 (117.2, 584.9)	0.6 (0.5, 8.1)
Jammu & Kashmir	89.1 (23.7, 289.3)	95.3 (27.0, 298.0)	4.4 (2.2, 8.6)	81.2 (18.2, 276.7)	87.7 (20.8, 285.1)	3.5 (1.9, 6.3)	62.49 (8.03, 234.89)	68.0 (10.9, 242.3)	2.0 (1.1, 3.4)	62.4 (8.0, 234.8)	68.0 (10.9, 242.3)	2.0 (1.1, 3.4)
Chhattisgarh	68.7 (40.1, 109.0)	69.4 (40.7, 109.6)	15.2 (4.3, 39.3)	42.5 (24.7, 75.5)	43.0 (25.2, 76.2)	10.6 (3.3, 23.7)	23.71 (12.28, 50.1)	24.0 (12.6, 50.6)	5.6 (2.4, 10.2)	23.7 (12.3, 50.2)	24.1 (12.6, 50.7)	5.9 (2.5, 10.2)
Delhi	5.3 (2.9, 10.0)	10.7 (7.8, 13.7)	4.6 (2.6, 8.3)	5.2 (2.9, 9.3)	9.7 (7.1, 12.6)	4.6 (2.6, 8.1)	4.92 (2.87, 8.61)	9.0 (6.6, 11.3)	4.4 (2.6, 7.6)	5.7 (3.2, 9.6)	9.0 (6.6, 11.3)	5.0 (3.0, 8.9)
Goa	22.3 (11.4, 36.6)	23.8 (12.9, 38.0)	3.9 (1.9, 11.4)	22.1 (11.3, 36.0)	23.7 (12.9, 37.3)	3.9 (1.9, 11.)	18.75 (10.2, 31.8)	19.8 (11.3, 33.4)	3.9 (1.9, 11.4)	21.9 (13.1, 34.5)	23.0 (14.2, 36.2)	7.4 (4.0, 14.6)
Haryana	25.9 (16.6, 36.5)	26.7 (17.5, 37.0)	9.2 (4.1, 19.3)	13.7 (8.3, 20.4)	14.2 (8.7, 20.8)	5.9 (3.0, 10.9)	6.68 (3.83, 11.91)	6.8 (4.0, 12.2)	3.6 (2.1, 6.0)	6.7 (3.8, 11.9)	6.8 (4.0, 12.2)	4.0 (2.1, 6.7)
Himachal Pradesh	138.0 (36.1, 464.7)	140.7 (36.7, 467.8)	9.5 (2.4, 24.3)	86.9 (19.2, 398.0)	89.3 (19.6, 401.0)	5.5 (2.1, 10.7)	71.18 (8.52, 379.28)	73.3 (8.8, 382.4)	3.19 (1.59, 6.13)	71.1 (8.5, 379.2)	73.3 (8.8, 382.4)	3.4 (1.8, 6.3)
Jharkhand	49.3 (30.5, 74.9)	50.2 (31.5, 75.8)	19.1 (5.0, 34.6)	28.4 (15.7, 49.4)	29.1 (16.4, 50.3)	6.2 (2.6, 14.2)	19.39 (9.27, 37.82)	20.0 (9.9, 38.6)	4.11 (1.9, 8.21)	19.4 (9.2, 37.8)	20.0 (10.0, 38.6)	4.3 (2.0, 8.3)
Tamil Nadu	27.4 (17.1, 40.3)	28.0 (18.0, 40.9)	7.1 (3.2, 19.6)	21.2 (12.8, 32.9)	21.8 (13.5, 33.6)	6.3 (3.1, 14.3)	7.21 (4.06, 13.39)	7.4 (4.2, 13.8)	3.84 (2.2, 6.07)	7.2 (4.0, 13.4)	7.4 (4.2, 13.9)	4.2 (2.3, 6.5)
Uttar Pradesh	36.7 (23.6, 53.2)	37.6 (24.8, 54.2)	16.0 (4.9, 32.8)	20.4 (11.6, 32.8)	21.1 (12.4, 33.7)	6.9 (3.1, 16.2)	12.51 (5.94, 21.64)	13.1 (6.3, 22.2)	4.3 (2.16, 8.84)	12.5 (5.9, 21.6)	13.1 (6.4, 22.2)	4.3 (2.1, 8.8)
West Bengal	37.9 (24.3, 53.5)	39.6 (26.4, 54.9)	23.1 (10.7, 37.2)	33.7 (21.1, 48.5)	35.5 (23.2, 49.7)	18.9 (9.2, 32.1)	12.14 (6.53, 20.57)	13.1 (7.1, 21.5)	6.64 (3.65, 11.21)	12.1 (6.5, 20.5)	13.1 (7.1, 21.5)	6.7 (3.7, 11.2)
Andhra Pradesh	56.8 (34.9, 83.6)	57.5 (35.6, 84.2)	19.4 (4.9, 40.3)	29.5 (17.7, 45.8)	29.9 (18.1, 46.1)	9.5 (3.4, 19.6)	13.63 (6.24, 24.83)	13.9 (6.4, 25.1)	3.72 (1.84, 6.74)	13.6 (6.2, 24.8)	13.9 (6.5, 25.1)	3.8 (1.9, 6.8)
Puducherry	5.5 (2.9, 9.2)	6.9 (4.4, 10.3)	2.6 (1.3, 4.2)	5.5 (2.9, 9.2)	6.9 (4.4, 10.3)	2.6 (1.3, 4.2)	4.04 (2.32, 5.98)	4.6 (3.3, 6.3)	2.32 (1.36, 3.88)	4.1 (2.4, 5.9)	4.6 (3.3, 6.3)	2.5 (1.5, 4.0)
Maharashtra	48.7 (32.7, 66.8)	49.3 (33.4, 67.2)	13.0 (4.0, 34.6)	37.0 (22.9, 54.3)	37.5 (23.6, 54.8)	8.9 (3.4, 24.)	12.69 (5.92, 21.77)	13.0 (6.1, 22.0)	4.13 (2.11, 7.04)	12.7 (5.9, 21.7)	13.0 (6.1, 22.0)	4.3 (2.1, 7.1)

**Table 7.** State-wise improvement in APC for a 30-minute time threshold.

State/UT	Rural				Urban				Total			
	Scenario I	Scenario II	Scenario III	Scenario IV	Scenario I	Scenario II	Scenario III	Scenario IV	Scenario I	Scenario II	Scenario III	Scenario IV
Andhra Pradesh	36.0	70.0	92.8	92.8	77.0	97.3	100.0	100.0	45.7	76.4	94.6	94.6
Arunachal Pradesh	30.9	48.6	53.3	53.3	93.2	100.0	100.0	100.0	33.4	50.7	55.2	55.2
Assam	44.0	67.6	84.9	84.9	78.0	91.5	97.5	97.5	49.9	71.7	87.1	87.1
Bihar	47.5	77.3	92.3	92.3	69.3	93.0	99.3	99.3	51.7	80.3	93.7	93.7
Chandigarh	100.0	100.0	100.0	100.0	99.9	99.9	99.9	99.9	100.0	100.0	100.0	100.0
Chhattisgarh	29.1	51.7	79.8	79.8	85.9	95.6	100.0	100.0	37.8	58.4	83.0	83.0
Dadra and Nagar Haveli and Daman and Diu	87.4	87.9	96.3	96.3	99.8	99.8	99.8	99.8	93.1	93.3	97.6	97.6
Delhi	96.2	96.2	96.2	96.2	100.0	100.0	100.0	100.0	100.0	100.0	100.0	100.0
Goa	93.7	93.8	95.6	92.4	94.0	94.0	94.0	94.0	93.5	93.6	94.9	92.6
Gujarat	41.0	72.5	93.6	93.6	90.4	96.6	99.3	99.3	57.8	80.4	95.0	95.0
Haryana	69.2	96.0	99.1	99.1	97.0	100.0	100.0	100.0	77.7	97.4	99.6	99.6
Himachal Pradesh	59.1	80.5	86.8	86.8	92.8	100.0	100.0	100.0	61.6	82.0	87.9	87.9
Jammu & Kashmir	62.2	67.1	72.8	72.8	96.5	97.6	98.4	98.4	68.6	72.8	77.6	77.6
Jharkhand	34.6	65.4	79.2	79.2	88.0	98.5	100.0	100.0	46.0	72.5	83.8	83.8
Karnataka	41.9	75.9	97.2	97.2	89.1	98.1	100.0	100.0	55.2	82.1	98.1	98.1
Kerala	94.3	95.4	96.4	96.4	98.2	98.2	98.2	98.2	95.9	96.4	96.9	96.9
Ladakh	23.8	31.6	37.7	37.7	71.8	71.8	71.8	71.8	23.9	31.7	37.7	37.7
Madhya Pradesh	26.0	46.2	81.9	81.9	82.9	92.5	100.0	100.0	35.7	54.2	85.2	85.2
Maharashtra	32.9	50.6	93.0	93.0	88.7	92.3	99.6	99.5	53.5	66.0	95.3	95.3
Manipur	60.3	64.3	74.6	74.6	100.0	100.0	100.0	100.0	70.9	73.9	81.5	81.5
Meghalaya	37.4	61.2	77.2	77.2	90.8	97.2	100.0	100.0	42.7	64.8	79.5	79.5
Mizoram	38.9	43.0	52.2	52.0	100.0	100.0	100.0	100.0	46.5	50.2	58.3	58.1
Nagaland	45.8	60.4	65.4	65.4	99.8	100.0	100.0	100.0	58.6	70.1	73.9	73.9
Odisha	28.5	67.5	79.7	79.7	84.0	100.0	100.0	100.0	36.2	71.9	82.6	82.6
Puducherry	96.1	96.1	96.1	96.1	99.0	99.0	99.0	99.0	97.7	97.7	97.7	97.7
Punjab	62.6	95.1	98.8	98.8	93.8	100.0	100.0	100.0	70.2	96.4	99.2	99.2
Rajasthan	23.1	71.6	90.5	90.5	74.5	98.8	100.0	100.0	32.3	76.5	92.4	92.4
Sikkim	86.4	87.4	89.2	89.2	100.0	100.0	100.0	100.0	87.5	88.3	90.0	90.0
Tamil Nadu	68.1	81.2	97.0	97.0	93.8	97.0	100.0	100.0	76.3	86.2	98.0	98.0
Telangana	38.2	63.4	94.2	94.1	91.6	97.6	100.0	100.0	55.6	74.5	96.3	96.3
Tripura	56.3	74.0	84.7	84.7	94.1	95.4	97.1	97.1	64.6	78.6	87.3	87.3
Uttar Pradesh	45.7	79.9	91.3	91.3	87.0	98.1	100.0	100.0	54.6	83.8	93.3	93.3
Uttarakhand	46.6	78.2	85.4	85.4	83.0	100.0	100.0	100.0	55.7	83.8	89.3	89.3
West Bengal	37.6	45.3	91.7	91.7	82.9	88.3	99.8	99.8	55.3	62.1	94.8	94.8

**Table 8.** State-wise improvement in APC for a 60-minute time threshold.

State/UT	Rural				Urban				Total			
	Scenario I	Scenario II	Scenario III	Scenario IV	Scenario I	Scenario II	Scenario III	Scenario IV	Scenario I	Scenario II	Scenario III	Scenario IV
Andhra Pradesh	73.2	95.2	97.8	97.8	95.3	100.0	100.0	100.0	78.4	96.5	98.5	98.5
Arunachal Pradesh	48.4	61.9	65.4	65.4	98.3	100.0	100.0	100.0	50.4	63.5	66.8	66.8
Assam	81.5	90.3	94.9	94.9	96.5	98.4	98.9	98.9	84.1	91.7	95.6	95.6
Bihar	91.4	97.0	98.5	98.5	98.5	100.0	100.0	100.0	92.8	97.6	98.9	98.9
Chandigarh	100.0	100.0	100.0	100.0	99.9	99.9	99.9	99.9	100.0	100.0	100.0	100.0
Chhattisgarh	63.8	85.5	94.1	94.1	96.8	100.0	100.0	100.0	68.9	87.9	95.2	95.2
Dadra and Nagar Haveli and Daman and Diu	96.1	96.5	96.6	96.6	99.8	99.8	99.8	99.8	97.5	97.7	97.8	97.8
Delhi	96.2	96.2	96.2	96.2	100.0	100.0	100.0	100.0	100.0	100.0	100.0	100.0
Goa	98.6	98.6	98.8	98.7	94.0	94.0	94.0	94.0	97.0	97.1	97.2	97.1
Gujarat	81.4	95.7	98.3	98.3	97.5	99.2	99.3	99.3	86.5	96.4	98.1	98.1
Haryana	98.3	99.6	99.6	99.6	100.0	100.0	100.0	100.0	99.0	100.0	100.0	100.0
Himachal Pradesh	83.9	91.1	92.5	92.5	100.0	100.0	100.0	100.0	85.1	91.8	93.2	93.2
Jammu & Kashmir	77.0	79.2	82.1	82.1	98.5	98.5	99.3	99.3	81.0	82.8	85.3	85.3
Jharkhand	75.3	90.0	94.5	94.5	99.8	100.0	100.0	100.0	80.5	92.4	95.9	95.9
Karnataka	81.5	98.2	99.3	99.3	97.9	100.0	100.0	100.0	86.1	98.8	99.7	99.7
Kerala	97.4	97.6	98.0	98.0	98.2	98.2	98.2	98.2	97.5	97.6	97.8	97.8
Ladakh	37.8	43.3	48.7	48.7	100.0	100.0	100.0	100.0	38.0	43.4	48.9	48.9
Madhya Pradesh	68.5	87.5	96.9	96.9	98.3	100.0	100.0	100.0	73.6	89.8	97.7	97.7
Maharashtra	77.9	88.8	98.4	98.4	98.5	99.3	99.6	99.6	85.4	92.5	98.7	98.6
Manipur	78.0	79.7	84.1	84.1	100.0	100.0	100.0	100.0	84.0	85.3	88.6	88.6
Meghalaya	74.5	87.5	92.3	92.3	98.6	100.0	100.0	100.0	76.9	88.7	93.1	93.0
Mizoram	60.8	64.0	70.2	70.1	100.0	100.0	100.0	100.0	65.9	68.6	74.1	73.9
Nagaland	72.0	77.8	80.9	80.9	100.0	100.0	100.0	100.0	79.1	83.5	85.9	85.9
Odisha	67.1	89.0	93.0	93.0	96.9	100.0	100.0	100.0	71.2	90.7	94.1	94.1
Puducherry	96.1	96.1	96.1	96.1	99.0	99.0	99.0	99.0	97.7	97.7	97.7	97.7
Punjab	98.6	99.2	99.2	99.2	100.0	100.0	100.0	100.0	99.1	99.6	99.6	99.6
Rajasthan	63.7	95.4	98.2	98.2	91.7	100.0	100.0	100.0	68.7	96.4	98.7	98.7
Sikkim	93.3	93.6	94.2	94.2	100.0	100.0	100.0	100.0	93.9	94.1	94.7	94.7
Tamil Nadu	96.5	97.6	98.6	98.6	100.0	100.0	100.0	100.0	97.7	98.4	99.1	99.1
Telangana	81.0	93.4	98.8	98.8	100.0	100.0	100.0	100.0	87.2	95.7	99.4	99.4
Tripura	83.8	89.7	94.0	94.0	96.9	97.6	98.4	98.4	86.6	91.4	94.9	94.9
Uttar Pradesh	89.2	96.4	98.2	98.2	99.6	100.0	100.0	100.0	91.4	97.3	98.7	98.7
Uttarakhand	83.1	90.8	92.5	92.5	99.4	100.0	100.0	100.0	87.2	93.3	94.6	94.6
West Bengal	85.5	89.7	98.1	98.1	98.1	99.0	100.0	100.0	90.3	93.3	98.8	98.8

**Table 9.** State-wise improvement in APC for a 120-minute time threshold.

State/UT	Rural				Urban				Total			
	Scenario I	Scenario II	Scenario III	Scenario IV	Scenario I	Scenario II	Scenario III	Scenario IV	Scenario I	Scenario II	Scenario III	Scenario IV
Andhra Pradesh	97.6	99.0	99.1	99.1	100.0	100.0	100.0	100.0	98.3	99.3	99.5	99.5
Arunachal Pradesh	69.3	72.6	75.1	75.1	100.0	100.0	100.0	100.0	70.5	73.7	76.2	76.2
Assam	97.1	98.0	98.5	98.5	98.9	99.0	99.0	99.0	97.4	98.1	98.6	98.6
Bihar	99.4	99.4	99.5	99.5	100.0	100.0	100.0	100.0	99.5	99.6	99.7	99.7
Chandigarh	100.0	100.0	100.0	100.0	99.9	99.9	99.9	99.9	100.0	100.0	100.0	100.0
Chhattisgarh	92.7	97.4	98.9	98.9	100.0	100.0	100.0	100.0	94.0	98.0	99.3	99.3
Dadra and Nagar Haveli and Daman and Diu	96.6	96.6	96.6	96.6	99.8	99.8	99.8	99.8	97.8	97.8	97.8	97.8
Delhi	96.2	96.2	96.2	96.2	100.0	100.0	100.0	100.0	100.0	100.0	100.0	100.0
Goa	99.1	99.1	99.2	99.2	94.0	94.0	94.0	94.0	97.4	97.4	97.5	97.4
Gujarat	97.6	99.0	99.1	99.1	99.3	99.3	99.3	99.3	97.6	98.5	98.6	98.6
Haryana	99.6	99.6	99.6	99.6	100.0	100.0	100.0	100.0	100.0	100.0	100.0	100.0
Himachal Pradesh	93.3	95.0	95.5	95.5	100.0	100.0	100.0	100.0	94.0	95.5	95.9	95.9
Jammu & Kashmir	86.6	87.2	89.0	89.0	100.0	100.0	100.0	100.0	89.1	89.5	91.1	91.1
Jharkhand	97.0	98.7	99.4	99.4	100.0	100.0	100.0	100.0	97.9	99.2	99.8	99.8
Karnataka	99.4	99.6	99.7	99.7	100.0	100.0	100.0	100.0	99.7	99.8	99.9	99.9
Kerala	98.7	98.7	98.8	98.8	98.2	98.2	98.2	98.2	98.2	98.2	98.3	98.3
Ladakh	51.6	55.4	60.2	60.2	100.0	100.0	100.0	100.0	51.7	55.5	60.3	60.3
Madhya Pradesh	98.2	99.2	99.6	99.6	100.0	100.0	100.0	100.0	98.8	99.6	99.9	99.9
Maharashtra	98.6	99.0	99.4	99.4	99.6	99.6	99.6	99.6	98.8	99.0	99.3	99.3
Manipur	88.6	89.2	90.7	90.7	100.0	100.0	100.0	100.0	91.8	92.3	93.4	93.4
Meghalaya	95.7	97.2	97.8	97.8	100.0	100.0	100.0	100.0	96.2	97.5	98.0	98.0
Mizoram	79.3	80.4	83.0	83.0	100.0	100.0	100.0	100.0	82.0	83.0	85.3	85.3
Nagaland	88.2	90.1	91.6	91.6	100.0	100.0	100.0	100.0	91.5	92.9	94.1	94.1
Odisha	94.3	97.7	98.8	98.8	100.0	100.0	100.0	100.0	95.2	98.1	99.0	99.0
Puducherry	96.1	96.1	96.1	96.1	99.0	99.0	99.0	99.0	97.7	97.7	97.7	97.7
Punjab	99.3	99.3	99.3	99.3	100.0	100.0	100.0	100.0	99.6	99.6	99.6	99.6
Rajasthan	95.3	99.4	99.5	99.5	100.0	100.0	100.0	100.0	96.3	99.7	99.8	99.8
Sikkim	96.0	96.0	96.3	96.3	100.0	100.0	100.0	100.0	96.3	96.4	96.7	96.7
Tamil Nadu	99.0	99.1	99.1	99.1	100.0	100.0	100.0	100.0	99.4	99.4	99.5	99.5
Telangana	98.7	99.2	99.6	99.6	100.0	100.0	100.0	100.0	99.3	99.7	99.9	99.9
Tripura	94.9	95.6	96.5	96.5	98.0	98.0	98.4	98.4	95.5	96.0	96.8	96.8
Uttar Pradesh	99.1	99.5	99.6	99.6	100.0	100.0	100.0	100.0	99.4	99.8	99.9	99.9
Uttarakhand	94.5	95.4	96.0	96.0	100.0	100.0	100.0	100.0	96.0	96.7	97.2	97.2
West Bengal	98.2	98.2	98.6	98.6	100.0	100.0	100.0	100.0	98.8	98.9	99.1	99.1

## Supplementary Table

**Supplementary Table 1.** Source of data and information extracted from each data source.

Data source	Information extracted
PI Directory [29]	List of PCC
PMGSY [22]	PHC
PMGSY	Community healthcare centers
NITI Aayog’s Report 2021 [23]	District hospitals
National Health Profile Report 2022 [24]	Teaching hospitals
Malaria Atlas Project (MAP) 2019 [25]	Data on motorized friction rasters
WorldPop dataset for 2020 [27]	High-resolution (1 sq. km) United Nations (UN) adjusted population counts
Meyers [28]	Indian national and state/UT administrative boundaries
